# BSE can propagate in sheep co-infected or pre-infected with scrapie

**DOI:** 10.1038/s41598-021-91397-8

**Published:** 2021-06-07

**Authors:** Angela Chong, James D. Foster, Wilfred Goldmann, Lorenzo Gonzalez, Martin Jeffrey, Matthew J. O’Connor, Keith Bishop, Ben C. Maddison, E. Fiona Houston, Kevin C. Gough, Nora Hunter

**Affiliations:** 1 The Roslin Institute, University of Edinburgh, Easter Bush, Midlothian, EH25 9RG UK; 2 Animal and Plant Health Agency Lasswade, Pathology Department, Pentlands Science Park, Bush Loan, Penicuik, Midlothian EH26 0PZ UK; 3 School of Veterinary Medicine and Science, The University of Nottingham, College Road, Sutton Bonington, Loughborough, Leicestershire LE12 5RD UK; 4 ADAS Biotechnology, School of Veterinary Medicine and Science, The University of Nottingham, College Road, Sutton Bonington, Loughborough, Leicestershire LE12 5RD UK

**Keywords:** Microbiology, Diseases, Pathogenesis

## Abstract

To understand the possible role of mixed-prion infections in disease presentation, the current study reports the co-infection of sheep with bovine spongiform encephalopathy (BSE) and scrapie. The bovine BSE agent was inoculated subcutaneously into sheep with ARQ/ARQ or VRQ/ARQ *PRNP* genotypes either at the same time as subcutaneous challenge with scrapie, or three months later. In addition, VRQ/VRQ sheep naturally infected with scrapie after being born into a scrapie-affected flock were challenged subcutaneously with BSE at eight or twenty one months-of-age. Sheep were analysed by incubation period/attack rate, and western blot of brain tissue determined the presence of BSE or scrapie-like PrP^Sc^. Serial protein misfolding cyclic amplification (sPMCA) that can detect very low levels of BSE in the presence of an excess of scrapie agent was also applied to brain and lymphoreticular tissue. For VRQ/ARQ sheep challenged with mixed infections, scrapie-like incubation periods were produced, and no BSE agent was detected. However, whilst ARQ/ARQ sheep developed disease with BSE-like incubation periods, some animals had a dominant scrapie western blot phenotype in brain, but BSE was detected in these sheep by sPMCA. In addition, VRQ/VRQ animals challenged with BSE after natural exposure to scrapie had scrapie-like incubation periods and dominant scrapie PrP^Sc^ in brain, but one sheep had BSE detectable by sPMCA in the brain. Overall, the study demonstrates for the first time that for scrapie/BSE mixed infections, VRQ/ARQ sheep with experimental scrapie did not propagate BSE but VRQ/VRQ sheep with natural scrapie could propagate low levels of BSE, and whilst BSE readily propagated in ARQ/ARQ sheep it was not always the dominant PrP^Sc^ strain in brain tissue. Indeed, for several animals, a dominant scrapie biochemical phenotype in brain did not preclude the presence of BSE prion.

## Introduction

Transmissible spongiform encephalopathies (TSEs) or prion diseases are fatal neurodegenerative conditions that occur naturally in various mammalian species, for example sheep (scrapie), deer (chronic wasting disease, CWD) and humans (e.g. Creutzfeldt Jacob Disease, CJD). TSEs are protein misfolding diseases but are unusual in that they are also transmissible between individuals, For instance, some TSEs can be transmitted through contact with bodily fluids^1^, via environmental contamination^2^, via contaminated feed/food^3^ or by experimental inoculation^4, 5^. With scrapie, the infectious agent, or prion, is readily detected in excreta and secreta^6–10^ and is widespread in the environment of such infected animals^11, 12^. The prion protein (PrP) is central to the pathogenesis of TSEs and exists in two main isoforms. PrP^C^ is a normal host protein which is expressed in many cell types especially in brain, and misfolded PrP^Sc^ which is so closely associated with TSE infectivity that it is believed to form part, or all, of the infectious agent^13^. Incoming PrP^Sc^ in an inoculum is believed to convert endogenous PrP^C^ into more PrP^Sc^ and hence to spread and replicate. PrP^Sc^ generated during disease progression is deposited in brain and lymphoid tissues and neuronal vacuolation is usually found at post mortem in infected brain. The protein conversion process (PrP^C^ to PrP^Sc^) can be mimicked in vitro including, in some cases, the creation of de novo infectivity, in a process termed serial protein misfolding cyclic amplification (sPMCA)^14, 15^.

TSEs are frequently studied experimentally in mice and sheep models from which considerable information has been generated over many decades on the transmission properties and characteristics of multiple prion strains. Strains can be defined in terms of incubation period (time between infection and disease)^16, 17^, interaction with particular host PrP (*PRNP*) genotype^5^, pathology, immunohistochemical (IHC) detection of patterns of PrP^Sc^ distribution^18^ and the appearance of the extracted PrP^Sc^ protein on western blots^19–21^. Strains are more usually studied singly in isolation where their characteristics are reproducible and predictable and therefore allow the study of particular aspects of early infection, pathology and pathogenesis. However, natural scrapie infections in sheep are not always due to a single strain and can be the result of mixtures of strains^22, 23^. Multiple passage in mice of isolates of natural scrapie can produce several different strains depending on the mouse lines used at each passage^22, 24, 25^. This has been interpreted as selection, from the original sheep classical scrapie infection, of particular strains that replicate more quickly or more efficiently in specific mouse lines, both inbred and transgenic^22, 24, 25^. Similar findings for CWD transmission in transgenic mice also suggest that deer infection might consist of more than one strain per outbreak^26^. Direct evidence for mixed infection within single sheep has been reported for classical scrapie and atypical scrapie^27, 28^, and the report of PrP^Sc^ in natural sheep scrapie with features akin to both bovine spongiform encephalopathy (BSE) and classical scrapie could be the result of mixed infection or a hybrid strain phenotype^29^.

Compared with the considerable literature on the biology of single TSE strain infections, much less has been reported on the effects of deliberately mixing strains. Because of the indirect methods used to identify strains, it is necessary to use two strains with easily identifiable features, for example short and long incubation periods, different patterns of brain vacuolation (lesion profiles) and those producing PrP^Sc^ with distinguishable patterns on western blots and/or differential antibody binding. In this way it has been established that a strain with a long incubation period can interfere with the ability of a short incubation period strain to produce disease^30, 31^. The strength of the interference is dependent on route of infection and precise timing but complete blockage of one strain by another can occur^32^. These studies gave rise to the idea that the number of replication sites is finite and that the binding of one strain will preclude binding of a second superinfecting strain. If large enough amounts of the first strain are used, the replication sites were postulated to be completely occupied and the superinfecting strain cannot then replicate^32^.

Additionally, strain interference was also demonstrated using the oral route suggesting it can occur in natural infections^33^ and evidence points strongly at PrP^C^ being the limiting resource (replication site) for which the strains compete^34^. Further studies in hamsters have also shown that although following some mixed infections all clinical disease and pathology seem to result from the faster replicating strain, more sophisticated tests (PrP^Sc^ stability) suggest that the slower replicating strain is still present, perhaps at low levels^23^.

Studies have shown that certain combinations of strain do not compete, and animals have the pathology and PrP^Sc^ of the shorter incubation strain without delay in incubation periods^35–37^. In addition, the two coinfecting strains can replicate independently, where an animal has the pathology of the shorter incubation strain without delay, but PrP^Sc^ is a mixture of both strains^23, 36–38^. Surprisingly, some animals can display a dominant PrP^Sc^ phenotype of a blocking strain yet the incubation period and clinical signs indicative of the faster strain^23^. In addition, the use of certain combinations of a non-transmissible strain with a highly pathogenic strain for coinfections can cause delay in the disease caused by the pathogenic strain. This suggests that either the non-pathogenic strain can replicate to some extent or that agent replication is not required for strain interference^36^. Overall, studies suggest that coinfections can lead to an inhibitory interference of one strain over another or independent replication of one or both strains.

Here, we present for the first time a study of mixed TSE infection in sheep. We used natural scrapie, SSBP/1 experimental scrapie and cattle BSE; the latter can be distinguished from the scrapie strains by incubation period, IHC and western blotting^39, 40^. Furthermore, a specific in vitro replication assay (sPMCA) that can detect BSE PrP^Sc^ even in the presence of a large excess of scrapie PrP^Sc^ was also applied^41, 42^. Sheep of different *PRNP* genotypes underwent co-challenge with BSE and scrapie (SSBP/1) or pre-infection with scrapie (either SSBP/1 or natural scrapie^43^) followed by BSE, and incubation periods were indicative of either scrapie or BSE disease. For selected animals, brain was analysed using western blotting to detect the dominant PrP^Sc^ type. Furthermore, brain and lymphoid tissue were analysed by a high sensitivity BSE-specific sPMCA assay. The objectives were to ascertain whether BSE will cause clinical disease when in competition with scrapie, and also to determine whether sheep displaying a scrapie phenotype in brain by conventional analysis (western blotting) could still harbour BSE prions.

## Results

### Incubation periods of sheep infected with scrapie and/or BSE

Cheviot sheep with VRQ/ARQ and ARQ/ARQ *PRNP* genotypes (referring to the three codons at positions 136, 154, 171) were injected subcutaneously with: SSBP/1 alone (S), BSE alone (B), SSBP/1 and BSE administered on the same day (S/B) or SSBP/1 followed after 3 months by BSE (S + B3m).

**Figure 1:**
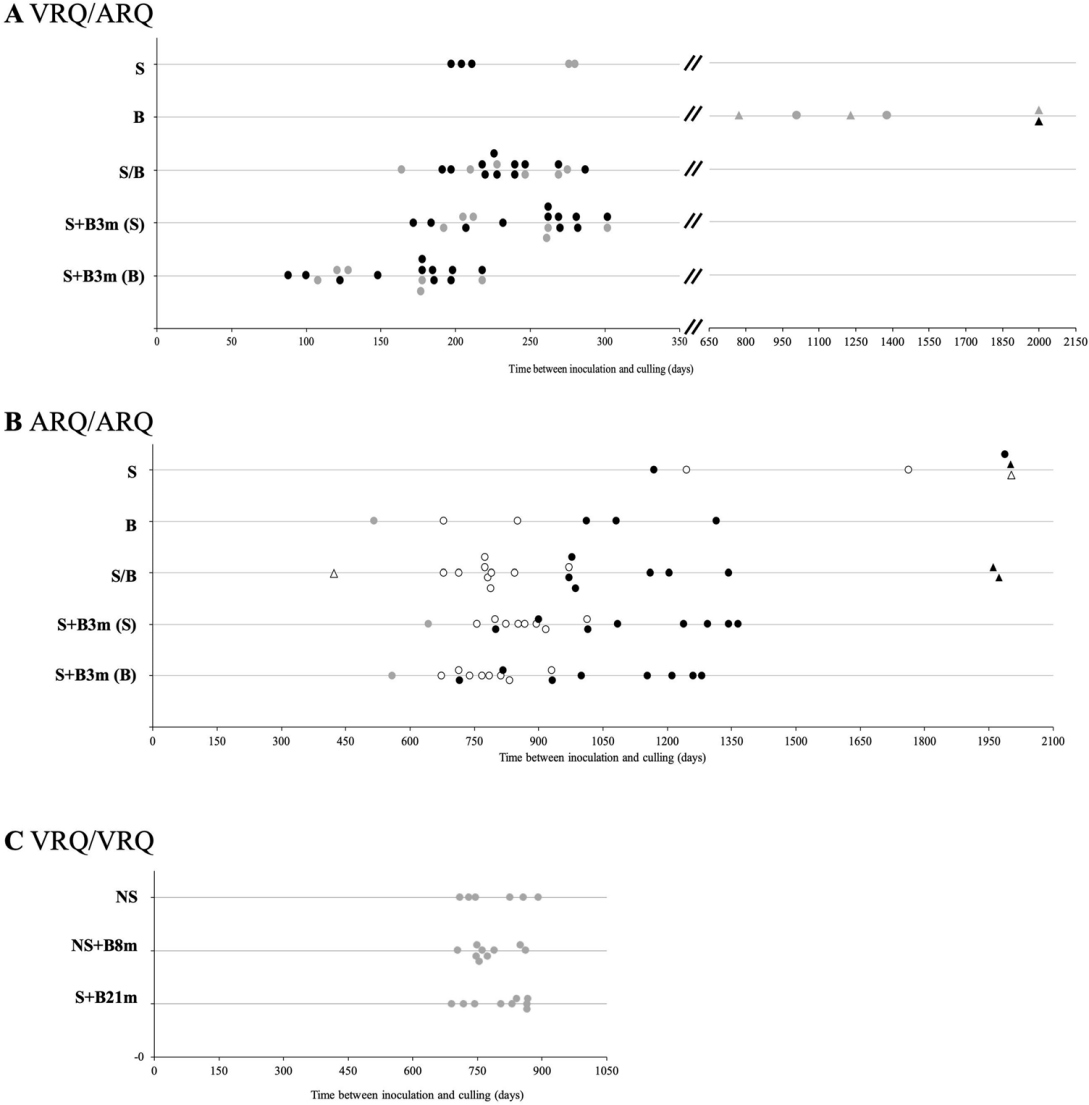
Incubation periods of individual VRQ/ARQ, ARQ/ARQ and VRQ/VRQ sheep. Sheep were inoculated with SSBP/1 scrapie, BSE or mixtures (**A**,**B**) or were infected with natural scrapie and then superinfected with BSE (**C**). The codon 141 *PRNP* genotype is indicated: LL_141_ (grey fill); LF_141_ (black fill); FF_141_ (no fill). Circles are clinical and pathology positive sheep, triangles are non-TSE animals. In **A** and **B**, lines represent SSBP/1, BSE, S/B, S + B3m dating from SSBP/1 inoculation (S), and S + B3m dating from BSE inoculation (**B**). In C, lines represent natural scrapie (NS) and NS superinfected with BSE at 8 months of age (NS + B8m) or 21 months of age (NS + B21m). For natural scrapie incubation periods are calculated from birth (exposure to scrapie).

The results of VRQ/ARQ sheep challenges are presented in Table 1 and Fig. 1. For SSBP/1 alone, all five inoculated sheep developed TSE clinical signs. The mean incubation period was 234 ± 41 days post injection (dpi). When challenged with BSE alone, only two out of six sheep developed TSE clinical signs with incubation periods of 1008 and 1229 dpi. Of the four sheep that did not develop clinical signs, two survived to the end of the study at 2001 dpi and two were culled with intercurrent disease (not TSE) at 774 and 1379 dpi.

**Table 1:** Incubation periods of TSE inoculated sheep.

*PRNP* genotype 136/154/171	Challenge group	Codon 141 subgroup	Number challenged	Clinically positive and PrP^Sc^ positive		Clinically negative and PrP^Sc^ negative	
				Number	Mean incubation period in days (± SD)^a,b,c^	Number	Survival times, range in days
VRQ/ARQ	SSBP/1 alone	All	5	5	234 (± 41)	0	
		LL	2	2	276, 280^d^	0	
		LF	3	3	204 (± 7)	0	
	BSE alone	All	6	2	1008, 1229	4	774–2001
		LL	5	2	1008, 1229	3	774–2001
		LF	1	0		1	2001
	S/B	All	17	17	233 (± 32)	0	
		LL	6	6	232 (± 41)	0	
		LF	11	11	233 (± 28)	0	
	S + B3m	All	17	17	S: 245 (± 42) B:161 (± 42)	0	
		LL	6	6	S: 239 (± 43) B: 155 (± 43)	0	
		LF	11	11	S: 248 (± 43) B: 164 (± 43)	0	
ARQ/ARQ	SSBP/1 alone	All	6	4	1541 (± 398)	2	2002, 2002
		LL	0				
		FF	3	2	1245, 1763	1	2002
		LF	3	2	1169, 1987	1	2002
	BSE alone	All	6	6	909 (± 289)	0	
		LL	1	1	516	0	
		FF	2	2	678, 851	0	
		LF	3	3	1136 (± 159)	0	
	S/B	All	17	15	918 (± 195)	2	1960^e^, 1975
		LL	0				
		FF	9	9	791 (± 83)	0	
		LF	8	6	1107 (± 153)	2	1960, 1975
	S + B3m	All	17	17	S: 998 (± 206) B: 914 (± 206)	0	
		LL	1	1	S: 643 B: 559	0	
		FF	8	8	S: 866 (± 79) B:782 (± 79)	0	
		LF	8	8	S: 1130 (± 213) B: 1046 (± 213)	0	
VRQ/VRQ	NS	All	6	6	NS: 793 (± 74)	0	
	NS + B8m	All	9	9	NS: 777 (± 50)	0	
	NS + B21m	All	9	9	NS: 803 (± 68)	0	

^a^S: time between scrapie inoculation and culling.

^b^B: time between BSE inoculation and culling.

^c^NS: time between birth (assumed exposure to scrapie) and culling.

^d^This animal had the P168L polymorphisms associated with longer incubation times for BSE.

^e^This animal had the M112T polymorphisms associated with longer incubation times for BSE.

All VRQ/ARQ sheep challenged with both SSBP/1 and BSE developed TSE clinical signs. S/B sheep (n = 17) had mean incubation period 233 ± 32 dpi. In the S + B3m group (n = 17), the data is presented in two ways. Firstly, the incubation period dating from the SSBP/1 inoculation, and secondly dating from the BSE inoculation. Mean incubation period for the whole group (n = 17) was 245 ± 42 from scrapie challenge (161 ± 42) dpi from BSE challenge). Comparing these results to those of the single inocula, the mixed infection groups had incubation periods indistinguishable from SSBP/1 infection (p > 0.99) and significantly different from those for BSE only (p < 0.001).

For VRQ/ARQ animals challenged with SSBP/1 only, there appeared to be shorter incubation periods for sheep with *PRNP* codon 141 genotype LF compared to LL_141_. However, numbers were low and inconsistent effects of this codon on incubation periods have previously been reported in similarly small cohorts of sheep challenged subcutaneously with SSBP/1^5^. Indeed, with the much larger mixed infections groups, incubation period did not appear to be influenced by the codon at 141 (p > 0.99 and p = 0.70 within S/B and S + B3m groups, respectively).

The incubation period results for the sheep of ARQ/ARQ *PRNP* genotype were in general much longer than those of VRQ/ARQ sheep (Table 1 and Fig. 1). For SSBP/1 alone, four out of the six sheep developed TSE clinical signs, with a mean incubation period of 1541 ± 392 dpi. There were two survivors at the end of the study at 2002 dpi. Although numbers are low, there was no evidence that codon 141 had any influence on incubation period (p = 0.89) as the four clinically positive animals included two each of FF_141_ (1245 and 1763 dpi) and LF_141_ (1169 and 1987 dpi). For BSE alone, all six challenged sheep developed TSE clinical signs, with mean incubation period of 909 ± 289 dpi. In this case the codon 141 genotype appeared to have an influence on incubation period length with the LF_141_ genotype having longer incubation periods (p = 0.03; Table 1, Fig. 1b).

In ARQ/ARQ sheep (n = 18) receiving a mixed inoculation (S/B), 15 developed clinical TSE signs with mean incubation period 918 ± 195 dpi indicative of a BSE incubation period. Although this group included no LL_141_ sheep, there was evidence that the FF_141_ homozygotes had shorter incubation periods than LF_141_ heterozygotes (mean 791 ± 83 and mean 1107 ± 153, respectively), although a slight overlap is present between groups (Fig. 1b; p = 0.002). Two further LF_141_ sheep remained healthy until they were culled at 1960 and 1975 dpi; one of these was heterozygous for the M112T *PRNP* polymorphism, which has previously been associated with resistance to TSEs^44^. A further animal was culled due to intercurrent disease at 421 days was also not included in the analysis.

In the S + B3m (n = 17), all sheep developed TSE clinical signs. Mean incubation period was 998 ± 206 days post scrapie challenge and 914 ± 206 days post BSE challenge, suggestive of BSE pathology. Again, the LF_141_ animals had longer mean incubation periods than LL_141_ and FF_141_ animals (p = 0.006), but again there was some overlap (Fig. 1b). Across all ARQ/ARQ animals that produced a BSE like incubation period, TSE disease incubation period was strongly influenced by the genotype at codon 141, with heterozygote LF_141_ sheep having extended duration to clinical disease (p < 0.001).

Comparing the results of mixed infections to those of the single inocula in ARQ/ARQ sheep, the mixed infection groups had incubation periods similar to BSE infection (p = 0.91 and p = 0.95 for S/B and S + B3m, respectively) and significantly different from those for scrapie (p < 0.001).

### Incubation times of natural scrapie sheep super-infected with BSE

Incubation time of VRQ/VRQ natural scrapie (NS) Roslin sheep (n = 6) was 793 d ± 74 until scrapie clinical signs developed. Of those animals which also received an inoculation of BSE at 8 m of age (NS + B8m, n = 9) clinical TSE signs developed and animals were culled with incubation time of 777 ± 50 days. In the group that received an inoculation of BSE at 21 m of age (NS + B21m, n = 9), incubation time was 803 ± 68 days. These incubation times are very similar to those of the control group showing no effect of the BSE super-infection (p = 0.69). Times of death following the BSE inoculation were 532 ± 53 days and 182 ± 68 days for NS + B8m and NS + B21m groups, respectively, and these times are likely to be too short to be BSE incubation periods or for the clinical signs to be the result of BSE rather than natural scrapie. We do not have data on subcutaneous inoculation of VRQ/VRQ sheep with BSE alone, however after intracerebral inoculation of VRQ/VRQ sheep with BSE, the average incubation period was > 1000 days^5^.

### Differential western blot analysis of PrP^Sc^ in brain from inoculated sheep

**Figure 2:**
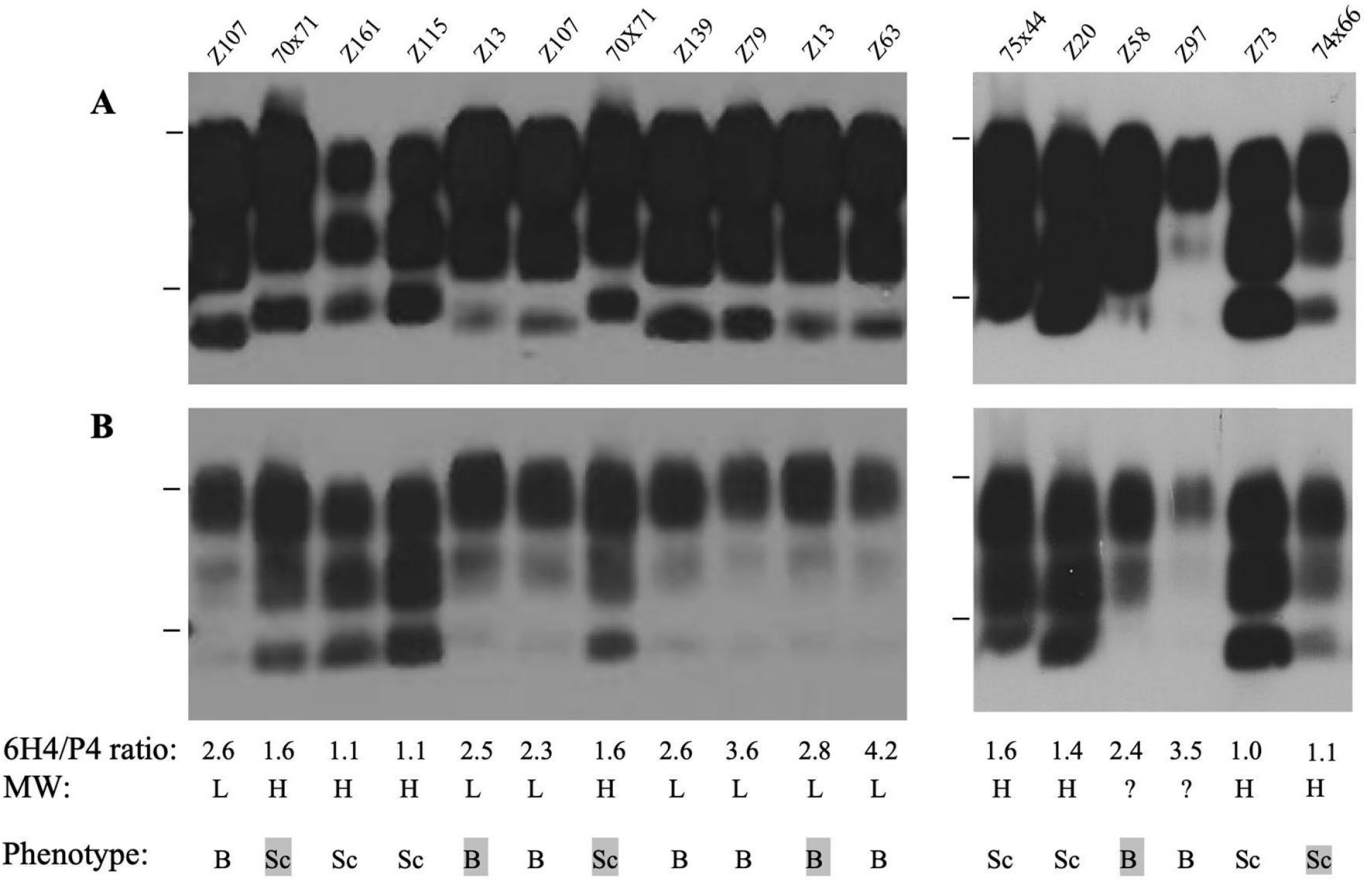
PrP^Sc^ phenotype in sheep brain. Brain homogenates were digested with PK and analysed on western blots probed with 6H4 (**A**) or P4 (**B**). Representative blots are shown. Approximate molecular weight (MW) indicated at 30 kDa and 20 kDa. Individual sheep references (e.g. Z107 or 70 × 71 formats) are indicated. A relatively low MW for the unglycosylated PrP band with 6H4 staining or a relatively low P4 signal (a ratio of 6H4/P4 signal of > 2) indicated the PrP^Sc^ had a BSE phenotype; strong P4 staining (a ratio of 6H4/P4 signal of 2 or less) and a relatively high MW unglycosylated band indicated a scrapie phenotype. These criteria and strain phenotypes are indicated. Shaded samples indicate sheep inoculated/infected with a single strain, all of these samples conformed to established molecular phenotypes of BSE or scrapie PrP^Sc^ and so provided within-blot controls for these phenotypes. Where samples were subjected to repeat analysis, the assay gave consistent results (examples shown for Z107, 70 × 71 and Z13).

The determination of the presence of BSE or scrapie-like PrP^Sc^ in brain was carried out by western blot analysis of proteinase K (PK) digested samples (Fig. 2, Supplementary Fig. S1, Table 2 for individual animals). BSE can be distinguished from scrapie on western blot by the slightly lower molecular weight of the unglycosylated PrP^Sc^ band (19 kDa vs 20 kDa). This reflects an altered PK cleavage site resulting in reduced staining by monoclonal antibody P4 in comparison to 6H4 in BSE samples, due to loss of the P4 epitope^39^. As expected, VRQ/ARQ sheep infected with SSBP/1 alone (n = 4) showed the typical scrapie-like PrP^Sc^ western blot profile, whilst the two VRQ/ARQ sheep infected with BSE alone displayed the BSE western blot profile (n = 2). VRQ/ARQ sheep infected with S/B or S + B3m (n = 10 in total) all produced a scrapie-like PrP^Sc^ pattern on western blot, in agreement with their incubation periods, which were as expected for SSBP/1 in this genotype. Similarly, ARQ/ARQ sheep infected with SSBP/1 alone (n = 2) showed scrapie-like PrP^Sc^ whereas those infected with BSE alone (n = 6) showed typical BSE-like PrP^Sc^ profiles. ARQ/ARQ sheep infected with S/B (n = 8) showed PrP^Sc^ patterns that were BSE-like, again in accordance with the incubation period data, which was typical of ARQ/ARQ sheep infected with BSE rather than SSBP/1. Six out of eight ARQ/ARQ sheep infected with S + B3m, gave a BSE-like PrP^Sc^ pattern on western blot. However, in two animals (Z73 and Z115) with the LF_141_ genotype, western blotting of PrP^Sc^ gave a scrapie-like signature.

**Table 2:** BSE-specific sPMCA test results compared with BSE and scrapie PrP^Sc^ determination by western blot analysis for individual sheep.

*Prnp* genotype	Inoculation group	Animal ID (codon 141 subgroup)^a^	Western blot^b^	BSE-specific sPMCA^c^		
			Brain^c^	Brain	Spleen	PSLN^d^
VRQ/ARQ	SSBP/1	Z75 (LF)	SC	NEG	NEG	NEG
		Z94 (LF)	SC	NEG	NEG	NEG
		Z26 (LF)	SC	–	–	–
		Z45 (LL)	SC	–	–	–
	BSE	Z48 (LL)	BSE	BSE	NEG	BSE
		Z65 (LL)	BSE	BSE	BSE	BSE
	S/B	Z92 (LL)	SC	NEG	NEG	NEG
		Z114 (LF)	SC	NEG	NEG	NEG
	S + B3m	Z66 (LF)	SC	NEG	NEG	NEG
		Z146 (LL)	SC	NEG	NEG	NEG
		Z161 (LF)	SC	NEG	NEG	NEG
		Z172 (LF)	SC	NEG	NEG	NEG
		Z121 (LF)	SC	–	–	–
		Z136 (LL)	SC	–	–	–
		Z49 (LL)	SC	–	–	–
		Z20 (LF)	SC	–	–	–
ARQ/ARQ	SSBP/1	Z10 (LF)	SC	NEG	NEG	NEG
		Z35 (FF)	SC	NEG	NEG	NEG
	BSE	Z13 (FF)	BSE	BSE	BSE	BSE
		Z28 (LF)	BSE	BSE	NEG	BSE
		Z37 (LL)	BSE	BSE	BSE	BSE
		Z43 (LF)	BSE	BSE	BSE	A
		Z53 (LF)	BSE	BSE	BSE	BSE
		Z58 (FF)	BSE	BSE	BSE	BSE
	S/B	Z79 (FF)	BSE	BSE	BSE	BSE
		Z91 (FF)	BSE	BSE	BSE	BSE
		Z97 (FF)	BSE	BSE	NEG	NEG
		Z113 (FF)	BSE	BSE	BSE	BSE
		Z116 (FF)	BSE	BSE	BSE	BSE
		Z88 (LF)	BSE	–	–	–
		Z107 (LF)	BSE	–	–	–
		Z63 (FF)	BSE	–	–	–
	S + B3m	Z54 (FF)	BSE	BSE	NEG	NEG
		Z90 (FF)	BSE	BSE	BSE	BSE
		Z115 (LF)	SC	BSE	BSE	BSE
		Z73 (LF)	SC	NEG	BSE	BSE
		Z64 (FF)	BSE	–	–	–
		Z44 (FF)	BSE	–	–	–
		Z59 (LF)	BSE	–	–	–
		Z139 (FF)	BSE	–	–	–
VRQ/VRQ	NS	74 × 66 (LL)	SC	NEG	NEG	NEG
		75 × 17 (LL)	SC	NEG	NEG	NEG
		70 × 71 (LL)	SC	–	–	–
	NS + B^e^	70 × 64 (LL)-21	SC	NEG	NEG	NEG
		72 × 35 (LL)-21	SC	NEG	NEG	NEG
		74 × 44 (LL)-8	SC	NEG	NEG	NEG
		75 × 09 (LL)-8	SC	NEG	NEG	NEG
		75 × 44 (LL)-8	SC	BSE	NEG	NEG
		74 × 90 (LL)-8	SC	–	–	–

^a^Animal codes are given (e.g. Z75).

^b^SC: Scrapie.

^c^NEG: negative for BSE by PMCA, –: not done, A: sample not analysed due to autolysis.

^d^PSLN: prescapular lymph node

^e^− 8 or − 21 indicate superinfection with BSE was carried out at 8 or 21 months of age, respectively.

In the natural scrapie sheep groups (n = 9), the PrP^Sc^ profile on western blot were all indicative of scrapie, regardless of whether or not they were also challenged with BSE.

### sPMCA analysis to detect BSE in inoculated sheep

During the study, a sPMCA method became available that can detect very low amounts of BSE PrP^Sc^ in a background of an excess of scrapie^41, 42^. The assay does not detect scrapie PrP^Sc^. Previous reports use the method with brain tissues, here it was applied to both brain, spleen and prescapular lymph node (PSLN) material. Initial analysis demonstrated that the use of LRS tissue did not have any matrix effects on the assay and the lowest level of ovine BSE brain spike that was analysed (a 10,000 × dilution of a BSE infected brain seed) was detected when diluted into brain or the LRS healthy tissues (data not shown).

**Figure 3:**
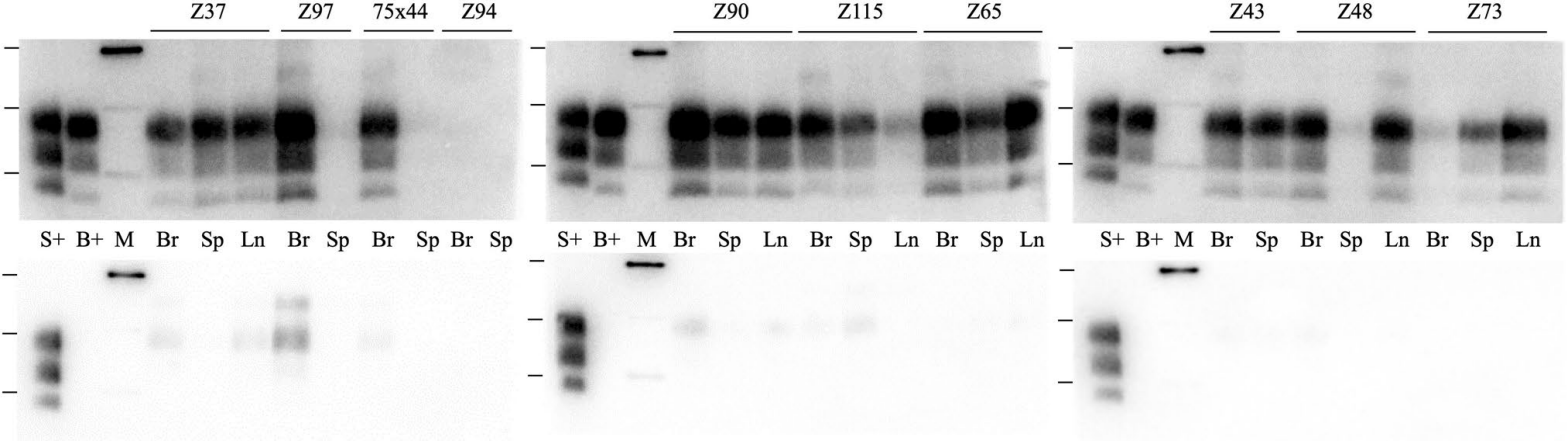
sPMCA amplification of BSE prions in sheep brain and LRS tissues. Following amplification of samples by sPMCA, PK-resistant PrP^Sc^ was detected with either SHa31 (top panels) or P4 (lower panels). Representative blots are shown. *Br *brain, *Sp* spleen, *Ln* PSLN tissue. Scrapie (S+) and BSE (B+) brain controls were also analysed on each blot. Z37 and Z43 were ARQ/ARQ sheep inoculated with BSE only; Z97 was an ARQ/ARQ sheep inoculated with S/B; Z90, Z115 and Z73 were ARQ/ARQ sheep inoculated with S + B3m; Z94 was a VRQ/ARQ sheep inoculated with SSBP/1 only; Z65 and Z48 were VRQ/ARQ sheep inoculated with BSE only; sheep 75 × 44 had natural scrapie and was superinfected, NS + B8m. Molecular weight markers (M) are shown at 20, 30 and 40 kDa and positions are also indicated on each blot.

Selected samples from each challenge group or control groups were analysed by sPMCA using both LRS and brain tissues; each animal had also been analysed by a differentiating western blot that could define a dominant scrapie or BSE PrP^Sc^ type in brain (Table 2, Fig. 3, Supplementary Fig. S2). The sPMCA assay did not detect BSE in any scrapie-only experimental challenge controls, as expected (4 × each of brain, spleen and PSLN samples). All samples from BSE-only challenged animals were positive for BSE with the exception of two spleen samples (from 8 × brain, 8 × spleen and 7 × PSLN samples). This may indicate that the BSE agent does not accumulate consistently within the spleen at clinical stages of disease. For experimental mixed infections (S/B, S + B3m) in sheep with VRQ/ARQ genotype, the sPMCA correlated with the western blot and incubation period data, in that all sheep examined (n = 6) had no detectable BSE PrP^Sc^ in brain, spleen or PSLN. For ARQ/ARQ sheep challenged with S/B (n = 5), BSE was detected in all brains and all but one animal had BSE present in the LRS samples analysed. The exception was Z97 that was negative for BSE in spleen and PSLN. In ARQ/ARQ sheep challenged with S + B3m, BSE was present in the brain and /or PSLN and spleen for all four animals analysed. For Z115, the sPMCA data showed BSE present in brain and LRS tissues and this was discordant with a dominant scrapie western blot in the brain. Similarly, Z73 was previously shown to have a dominant scrapie brain PrP^Sc^ profile in western blots and whilst the brain was also negative for BSE by sPMCA, BSE could be detected in spleen and PSLN.

For NS infection, no BSE was found in scrapie-only controls (n = 2), as expected. In the animals superinfected NS + B21m (n = 2), no BSE was present in brain or LRS. When BSE superinfection was NS + B8m (n = 3), all samples examined were negative for BSE, except the brain sample from animal 75 × 44. The western blot profile for this brain indicated scrapie PrP^Sc^ was dominant.

### Immunohistochemistry analysis of tissue from sheep demonstrating a dominant scrapie western blot profile in brain but also propagating BSE in the LRS

**Figure 4:**
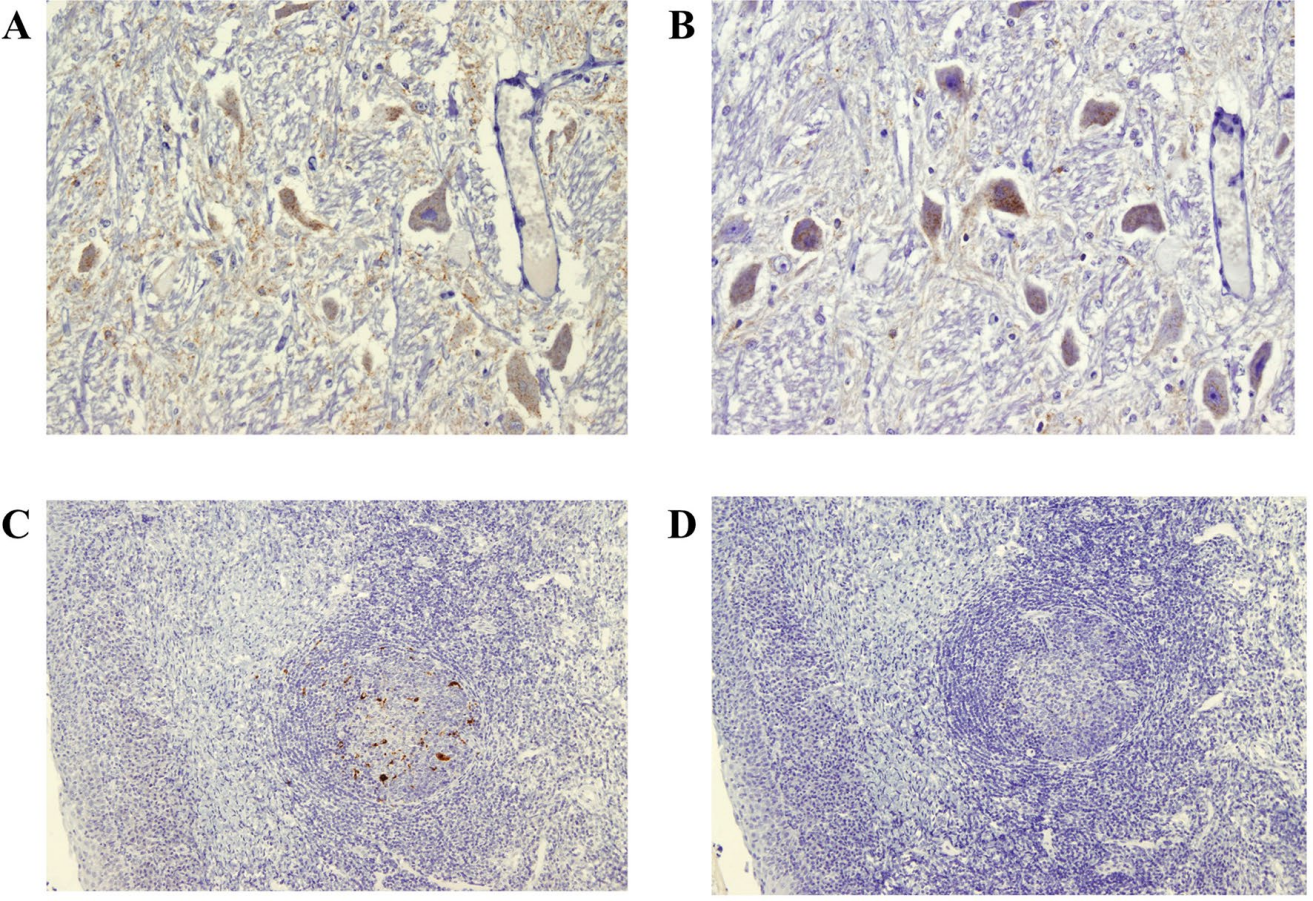
Discriminatory immunohistochemistry of sheep brain and tonsil from a mixed infection sheep with scrapie signal in brain and BSE in periphery. Sections of medulla [lateral cuneate nucleus; (**A**, **B**); × 20] and tonsil [(**C**,**D**); × 10) from VRQ/ARQ sheep Z115 were stained with antibody R145 in (**A**) and (**C**); or 12B2 in (**B**) and (**D**). Results show labelling of intraneuronal PrP^d^ with both R145 and 12B2 in brain (**A**,**B**), whereas in tonsil (**C**,**D**) labelling of tingible body macrophages within germinal centre was seen only with R145, the 12B2 reaction being negative.

Previous work has shown that BSE and scrapie can be distinguished on IHC by including certain antibodies (e.g. 12B2) that label intracellular PrP^d^ (intraneuronal in brain, in tingible body macrophages of lymphoid tissues) in scrapie-infected, but not BSE-infected sheep^40^. This method was therefore applied to samples from the two animals in the S + B3m group that demonstrated discordant data in western blot and sPMCA, Z115 and Z73. Both demonstrated a dominant scrapie phenotype in brain by western blot but the presence of BSE prion in brain and/or LRS by sPMCA. Tissues for animal Z73 could not be reliably analysed due to autolysis. IHC data for Z115 confirmed that the dominant TSE type in brain was scrapie whilst that in LRS (tonsil) was BSE (Fig. 4).

## Discussion

There are relatively few reports on the study of mixed prion infections. Rodent models have demonstrated that experimental infection with two prion strains can result in the dominance of the strain with the most favourable replication properties such as a shorter incubation time. In these circumstances this dominant strain can cause pathology with correlating PrP^Sc^ phenotype accumulation at the end stage of disease^36, 38, 45^. However, models have also shown that the slower replicating strain can delay or completely block the disease of the faster strain^33, 35, 46^. The delayed disease then usually has the clinical presentation and PrP^Sc^ of the faster strain. Alternatively, mixed infections can result in independent replication of both strains^37^.

This is the first study on experimentally mixed infections in sheep. There has been considerable historical concern that the BSE agent may have propagated in sheep and been masked by the scrapie agent. This was a particular concern in the UK where endemic scrapie existed and it was considered that sheep were probably exposed to BSE-contaminated feed during the bovine BSE epidemic (Statement by Spongiform Encephalopathy Advisory Committee: “Experimental transmission of BSE to sheep and risk of exposure to BSE through feed (10/07/1996). Archived at: https://webarchive.nationalarchives.gov.uk/20070308092252/http://www.seac.gov.uk/statements/state10jul96.htm). However, considerable surveillance has failed to detect any BSE cases in sheep and only two from goats, despite sheep being susceptible to bovine BSE through experimental oral challenge^47^. This raises the questions of how coinfection of scrapie and BSE compete or co-exist within a ruminant host. To investigate this, we carried out mixed infection studies in sheep with scrapie and ovine BSE. The study looked at sheep challenged with both TSEs simultaneously and also when challenged with BSE three months after scrapie. In addition, sheep naturally infected with scrapie were challenged with BSE at eight and 21 months of age.

VRQ/ARQ sheep propagate SSBP/1 scrapie with a mean incubation period was 234 ± 41 days post injection (dpi), which compares well with previous studies^48^. This disease progression is more effective and more rapid (approximately 5 × shorter incubation period) than BSE. Challenge with BSE alone resulted in only two out of six sheep developing TSE clinical signs with incubation periods of 1008 and 1229 dpi. In contrast, in a previous study, VRQ/ARQ (LL_141_) sheep infected with BSE by the intracerebral route had average incubation periods of 875 ± 77 dpi and a 100% attack rate^5^. Data illustrates that infection with BSE by subcutaneous inoculation is less efficient than intracerebral inoculation. Mixed infections of such sheep with both strains resulted in a rapid progression to clinical disease and only scrapie PrP^Sc^ could be detected in brain or LRS by western blotting in animals that were tested, and sPMCA failed to detect low levels of BSE PrP^Sc^. These data indicate that the VRQ/ARQ sheep died from scrapie infections before the BSE agent could accumulate to a detectable level and that the BSE agent did not compete with the scrapie agent when animals were infected with both strains. This supports the conclusion that challenge of sheep with BSE did not interfere with scrapie disease progression in a genotype where scrapie is the faster replicating strain.

The propagation of SSBP/1 and BSE in ARQ/ARQ sheep presents a different scenario, where BSE propagates more efficiently and slightly more rapidly (~ 1.7 × shorter incubation period) than scrapie. For SSBP/1 alone, four out of the six sheep developed TSE clinical signs, with a mean incubation period of 1541 ± 392 dpi, with two survivors at the end of the study at 2002 dpi. This prolonged incubation period is similar to a previous study that challenged ARQ/ARQ sheep with SSBP/1 and reported no clinical disease at 1108 days^48^. For BSE alone, all six challenged sheep developed clinical disease with mean incubation period of 909 ± 289 dpi. For challenge with both TSEs at the same time, the incubation periods were more reflective of BSE infection but the attack rate was incomplete. Western blot and sPMCA showed that the PrP^Sc^ present in brain and LRS was BSE. Combined, data indicates the animals in this cohort likely all died from the BSE agent that was the dominant PrP^Sc^ in the tissues analysed. For delayed challenge with BSE after scrapie inoculations, the outcomes were more complex. The incubation periods were relatively rapid and with high attack rates indicating BSE infections. Biochemical analysis of tissues showed that most sheep displayed dominant BSE PrP^Sc^ in all tissues analysed. However, two sheep had a dominant scrapie PrP^Sc^/PrP^d^ phenotype in brain (examined by western blot and/or IHC) despite displaying incubation periods most indicative of BSE infection. These animals also had BSE agent present in LRS tissues and/or brain when analysed by sPMCA. This data correlates with a small number of individual animals in a rodent model of prion superinfections where they displayed a dominant PrP^Sc^ phenotype of the slower blocking strain yet the incubation period and clinical signs were indicative of the faster strain^23^. There is no assay available that can detect very low levels of scrapie PrP^Sc^ in the presence of high levels of BSE PrP^Sc^ and so it is not known whether the ARQ/ARQ sheep with BSE pathology and a dominant BSE PrP^Sc^ phenotype harboured low levels of scrapie PrP^Sc^.

When considering the polymorphism at position 141, this did not appear to have any influence on the incubation periods for VRQ/ARQ sheep where scrapie pathology was dominant. In contrast, in ARQ/ARQ sheep where BSE-like incubation periods were observed, the LF_141_ genotype had significantly longer incubation periods than homozygous genotypes. A similar effect has been previously reported for BSE infections in ARQ/ARQ sheep^49^. The small number of ARQ/ARQ sheep infected with SSBP/1 only did not show any evidence that codon 141 influenced incubation period.

For natural scrapie infections followed by BSE challenge, all animals displayed scrapie like PrP^Sc^ in brain by western blot. No amplifiable BSE PrP^Sc^ could be detected in spleen or PSLN by sPMCA. However, one of five brain samples analysed by sPMCA showed the presence of BSE even though the dominant western blot signature was scrapie-like. Again, this demonstrates the replication of multiple prion strains within a ruminant host and the masking of the presence of BSE prion by a dominant scrapie pathology.

Whilst this was not an exhaustive study of ovine genotypes and their susceptibility to mixed infections, the study demonstrates that in sheep, a rapid TSE strain usually ‘out competes’ a slower propagating strain and causes the pathology/clinical disease. However, the data also shows that the dominant PrP^Sc^ does not always correlate with this pathology, for instance scrapie can be the most dominant PrP^Sc^ in brain even though incubation periods would indicate a BSE disease. Furthermore, the dominant PrP^Sc^ strain found in brain does not preclude the presence of another co-infecting strain. Here, of thirteen co-infected sheep that displayed a scrapie-like biochemical phenotype in brain by western blot analysis and were also analysed by sPMCA, three had detectable BSE in brain and/or LRS. This study therefore highlights that surveillance for specific TSE strains requires analyses of distinct tissues with a suite of biochemical tests to fully elucidate their presence or likely absence. This is significant as strains present at low levels within mixed infections could potentially become the dominant, disease-causing agent due to changes in the replication environment, for example sub-passage into a host with a different *PRNP* genotype.

## Methods

### Sheep inoculations

For experimental infections, Cheviot sheep were sourced from the Defra flock of New Zealand origin that was free of classical scrapie. The sheep genotypes were confirmed as previously described^47^ (eg VRQ/ARQ). After the project was initiated, and animal groups chosen, additional sequencing detected variation at other codons (112, 141 and 168) on the ARQ alleles encoded by the sheep. Numbers of animals with codon 112 or 168 variants were too small to be informative (single animals), however codon 141 variation (L replaced with F) was much greater, although in uneven proportions, and is presented here as a genotype at codon 141 alone (LL, FF or LF in Tables, or e.g. LL_141_ in text). The other relevant sheep *PRNP* allele is VRQ, which shows no variation at codons 112, 141 or 168.

Sheep of genotypes VRQ/ARQ and ARQ/ARQ were challenged with the well characterised scrapie brain pool, SSBP/1^48^ and/or with cattle BSE, an inoculum termed BBB/2/02 made by pooling six cattle brains supplied by the Animal and Plant Health Agency (APHA) and used previously in other sheep challenge studies^50^. Animals were injected subcutaneously in the medial thigh with 10% (w/v) homogenates of SSBP/1 or BSE alone or together in mixed inoculations. Sheep were challenged with either SSBP/1 or BSE by injection of two separate 1 ml doses (2 ml total dose) into each hind leg. The mixed injection groups, herein described as S/B, were each injected with 2 ml of a 1:1 by volume mixture of SSBP/1 and BSE homogenates in each hind leg. The separate injection groups, herein described as S + B3m, were first injected with 2 × 1 ml SSBP/1, and 3 months (84 days) later with 2 × 1 ml BSE in each hind leg. All sheep were between 789 and 878 days old at the primary challenge.

To investigate the interaction of BSE and natural scrapie, Cheviots were sourced from the Roslin Scrapie Flock (NPU Cheviot Flock). Natural scrapie is endemic in these sheep affecting 100% of VRQ/VRQ sheep at around 2 years of age. Infection is assumed to occur in these animals at or around the time of lambing but the timing is not known precisely. Incubation times for disease in these animals is calculated from birth. It is likely that by the time the sheep were exposed to BSE, natural scrapie infection would have been well established. This part of the study aimed to replicate more closely a realistic situation whereby a sheep incubating natural scrapie might become superinfected with BSE by peripheral infection. A control group of VRQ/VRQ sheep was left to develop natural scrapie with no additional challenge and are described as NS sheep. Additional animals were inoculated subcutaneously with BSE as above (2 × 1 ml of 10% BSE cattle brain homogenate into the hind limbs). These animals are described as NS + B. Animals were either challenged with BSE at 8 months of age (NS + B8m; age at challenge was between 234 and 258 days) or were challenged at 21 months of age (NS + B21m; age at challenge was between 614 and 633 days), the latter time point is just prior to the expected development of natural scrapie clinical signs.

All of the animals were observed daily for clinical signs of TSE and culled humanely once such signs were clearly seen at agreed end-points. Any animals with intercurrent illness were treated by a veterinary surgeon and culled humanely if recovery was not apparent. All animals were subject to post mortem collection of brain and lymphoid tissues, for subsequent analysis. Times between inoculation and culling were recorded as follows. An incubation period was assigned when an animal had both TSE clinical signs and positive PrP^d^ detection by IHC analysis of brain. A survival time was assigned when an animal was negative for one or both of these features (TSE signs and PrP^d^). Sections of the brainstem at the level of the obex and the thalamus from all sheep, including intercurrent deaths, were labelled by IHC using the monoclonal antibody BG4 (binding epitopes at 47–57 and 89–99 in bovine PrP), in order to achieve a positive or negative confirmation of disease status (data not shown but outcome is implicit in the reported incubation periods).

All sheep studies were reviewed and approved by Animal Welfare and Ethical Review Committees at IAH Compton and the Roslin Institute and carried out in accordance with the Animals (Scientific Procedures) Act 1986, under the authority of UK Home Office Project and Personal Licenses (and complying with the ARRIVE guidelines).

Statistical analysis of incubation period data was carried out using one way ANOVA followed by Tukey’s multiple comparison analysis when comparing incubation times between challenge groups. When comparing coinfection groups to BSE only challenge, the incubation times from BSE challenge were used; when comparing coinfection groups to scrapie only challenge, the incubation times from scrapie challenge were used. When comparing incubation periods between sheep homozygote or heterozygote for codon 141, an unpaired Student’s T test was used.

### Detection of BSE or scrapie PrP^Sc^ by western blotting

Selected samples were subjected to PrP^Sc^ analysis by PK digestion and western blot analysis as previously described^39, 40^. Samples were run on 16% (w/v) Tris–glycine gels (Invitrogen, Paisley, UK) and immunoblotted onto polyvinylidene difluoride (PVDF) membranes. For detection of sheep PrP^Sc^, two monoclonal antibodies were used: 6H4 (epitope 144–152 in human PrP, Prionics, Schlieren, Switzerland, 2 mg/ml, diluted 1:5000), and P4 (epitope 93–99 in ovine PrP; R-Biopharm, Darmstadt, Germany, 1 mg/ml, diluted 1:2500). The "visualisation" process used a chemiluminescence substrate (Roche, Lewes, UK) and Lumi-film (Roche). For re-probing a membrane with a second antibody, the 6H4 antibody was removed using Restore (Thermo Fisher Scientific, Loughborough, UK) and then the membrane was re-probed with the P4 antibody. Each blot was imaged at 3 different exposure times (30 s, 3 min and 10 min) to allow visualisation of the unglycosylated band. The molecular weight of this band was determined by comparison to the MW markers and was assigned as relatively low or high with a difference of ~ 2 kDa. Densitometry analysis (using Image J) of blots allowed identification of the ratio of PrP^Sc^ signals after probing with each antibody, a ratio of 6H4/P4 signal greater than 2 indicated BSE, analysis criteria are as previously described^39^. Samples were defined as BSE or scrapie when they consistently met both MW and relative P4 reactivity criteria. However, some ovine BSE samples could not be resolved to determine the MW of the unglycosylated band (due to very low levels of this form of PrP^Sc^), in these cases the definition of BSE was confirmed by the P4 reactivity criteria alone.

### Serial PMCA to detect BSE PrP^Sc^

Selected sheep tissues were examined using sPMCA optimised for the specific detection of BSE as previously described^41^. This technique scores samples as positive or negative for BSE and does not detect scrapie. In summary, tissue (100–150 mg) was homogenised in lysis buffer (PBS containing 0.5% (w/v) sodium deoxycholate and 0.5% (v/v) NP40 to give a final 10% (w/v) tissue) using a mini beadbeater set at 4800 rpm for 60 s (brain and spleen) or 180 s (PSLN). Homogenised samples were aliquoted and frozen at − 80 °C. sPMCA amplification was carried out for 5 rounds in total using alternating AHQ/AHQ and VRQ/VRQ sheep brain substrates for each round. For amplifications, two different sPMCA sonicator horns were each used to analyse a replicate of the same sample. A sample was noted as being BSE positive if either replicate produced PrP^Sc^ with a BSE western blot phenotype. During the sPMCA, samples were diluted 1:3 every 24 h with the substrate, as previously described^41^. An equal number of test samples and scrapie only (negative control) samples were amplified on each sPMCA run. No negative controls produced any PrP^Sc^ signal (data not shown).

Following sPMCA, samples were digested with 50 µg/ml PK at 37 °C for 1 h in the presence of 0.5% (w/v) SDS. An equivalent to 5 µl of the original PMCA reaction was then subjected to SDS PAGE using a 12% (w/v) NuPAGE electrophoresis system with MOPs buffer. The separated proteins were electroblotted to PVDF membrane and blocked with 3% (w/v) milk protein overnight. Duplicate blots were probed with the monoclonal antibody SHa31 (epitope 145–151 in hamster PrP) or P4. Densitometry analysis of blots probed with SHa31 allowed identification of samples that produced a PrP^Sc^ signal above the background produced with control scrapie samples. Samples were scored positive for BSE when producing a SHa31 signal above background and a ratio of their SHa31/P4 signal greater than 2.9, analysis criteria are as previously described^41, 42^. We have previously demonstrated the limit of detection of BSE by sPMCA in the presence of scrapie-infected brain^41^. The assay gave 100% sensitivity at 1/50 dilution of BSE in scrapie, falling to 90% and 80% sensitivity at 1/225 and 1/500 dilutions, respectively. Here, a demonstration of the assay limit of detection was again performed, to further evaluate any effects of the spleen and PSLN matrix with respect to the amplification of BSE. These experiments were carried out twice using two different sPMCA sonicators. Due to delays in the availability of spleen tissue from TSE-free sheep, these experiments were carried out in a background of healthy ovine brain and PSLN samples and the spleens of tg338 mice (mice expressing ovine VRQ PrP)^51^.

### Detection of BSE or scrapie PrP^d^ by immunohistochemistry

The IHC methods do not result in detection of PrP^C^ but as no PK is used in the process, the PrP protein detected by IHC is referred to here as disease-associated PrP (PrP^d^)^40^. The term PrP^Sc^ is reserved for the protein detected in PK treated samples.

Brain and lymphoid tissues were analysed using a method that discriminates between BSE and scrapie and has been described in detail several times previously^18, 40^. When antibodies that recognise specific epitopes within the N terminus of PrP are used, epitopes to the sequence 93–99 of the PrP protein are preserved in SSBP/1 but lost in BSE^40^. Labelling was with R145 (epitope 221–232 in bovine PrP) and the N-terminal discriminatory antibody 12B2 (epitope 93–97 in bovine PrP).

## Supplementary Information

Supplementary Information.

## Data Availability

The datasets generated during and/or analysed during the current study are available from the corresponding author on reasonable request.
